# Influence of Test Method on the Determination of Tensile Strength Perpendicular to Grain of Timber for Civil Construction

**DOI:** 10.3390/ma17184506

**Published:** 2024-09-13

**Authors:** Rodrigo de Souza Nogueira, Fabiana Yukiko Moritani, André Luis Christoforo, Sergio Neves Monteiro, Afonso Rangel Garcez de Azevedo, Herisson Ferreira dos Santos, Francisco Antonio Rocco Lahr

**Affiliations:** 1 Department of Structural Engineering, São Carlos School of Engineering, University of São Paulo, Av. Trabalhador São Carlense, 400 Centro, São Carlos 13566-590, SP, Brazil; frocco@sc.usp.br; 2 Department of the Civil Engineering (FCTUC), University of Coimbra, Rua Luís Reis Santos-Pólo II, 3030-788 Coimbra, Portugal; fabianamoritani@gmail.com; 3 Department of the Civil Engineering—UFSCAR, Federal University of São Carlos, Rodovia Washington Luís, km 235-SP-310, São Carlos 13565-905, SP, Brazil; christoforoal@yahoo.com.br; 4 Materials Science Department, Military Institute of Engineering (IME), Praça General Tibúrcio, 80 Urca, Rio de Janeiro 22290-270, RJ, Brazil; sergio.neves@ime.eb.br; 5 Civil Engineering Laboratory (LECIV), Science and Technology Center, North Fluminense State University Darcy Ribeiro, Av. Alberto Lamego, 2000-Parque Califórnia, Campos dos Goytacazes 28013-602, RJ, Brazil; afonso@uenf.br; 6 Campus of Ariquemes, Federal Institute of Education, Science and Technology of Rondônia (IFRO), Av. Juscelino Kubitschek, 2717-2853 Setor Industrial, Ariquemes 76870-000, RO, Brazil; herisson.santos@ifro.edu.br

**Keywords:** tensile perpendicular to grain, three-point bending test, uniaxial tensile test, species from planted forests, nonlinear regression models

## Abstract

Tensile perpendicular to grain is an important mechanical property in the design of joints in timber structures. However, according to the standards, this strength can be determined using at least two different methods: uniaxial tensile and three-point static bending. In this context, the present paper aims to investigate the influence of these test methods on the determination of tensile strength perpendicular to grain of wood used in civil construction timber. Three wood species from Brazilian planted forests (*Pinus* spp., *Eucalyptus saligna*, and *Corymbia citriodora*) were used in this investigation. Twelve specimens of each species were used for each test method investigated. Moreover, a statistical analysis was performed to propose an adjustment to the equation of the Code of International Organization for Standardization 13910:2014 for the three-point bending test. Tensile strength values perpendicular to grain obtained from the uniaxial tensile test were significantly higher than those determined by the three-point bending test. It is proposed that the tensile strength perpendicular to grain can be determined more precisely with adoption of coefficient 5.233 in the term [(3.75·Fult)/b·Lh] of the equation specified by the Code of International Organization for Standardization 13910:2014 for the three-point bending test.

## 1. Introduction

Building materials most commonly used in structural systems are concrete, steel, timber, and masonry (brick or block). However, timber is gaining more attention due to its desirable properties related to sustainability, energy efficiency, speed of construction, and high seismic capacity [1]. In addition, timber contributes to the environment preservation through carbon mitigation. Indeed, carbon is sequestered from atmosphere during the tree’s development, and only a portion of the carbon is lost to atmosphere once the tree and its wooden products reach the end of their usage [2]. Timber, as a building material, has a higher strength-to-density ratio than reinforced concrete and steel [3,4]. Moreover, timber also exhibits a lower energy-to-strength ratio compared to reinforced concrete and steel, with the advantage that the energy used for growing trees comes from sunlight [3].

Planted forests with introduced species, such as pine trees in Brazil, can contribute to carbon sequestration and supply raw materials for civil construction. Globally, planted forests in 2020 represented about 7% of the total forest area (4.06 billion ha) [5]. In South America, 97% of the planted forests in 2020 consisted of introduced species [5]. According to Brazilian Tree Industry (IBÁ) [6], in 2018, *Pinus* and *Eucalyptus* were the genera most cultivated in Brazilian country. In addition, these two genera represented 92.46% of the total planted forest area in Brazil [6]. Therefore, knowledge of mechanical properties of timber is essential for its application as a structural element in construction. Thus, research works were developed in order to characterize the mechanical properties of various wood species [7,8,9,10,11,12].

Timber exhibits different mechanical properties among its wood grain directions as a result of its anisotropy. However, criteria codes for timber structures are based on load position relative to wood grain in structural elements [13,14]. An important mechanical property in the design of timber structures, which can lead to their collapse due to local stresses perpendicular to grain at certain joints, is the tensile perpendicular to grain [15]. Such failure may occur at a lower force level in joints of joist hangers, glued-in bolts, punched metal plate fastener joints and joints with dowels or rings, as well as shear plate connectors [15]. In addition, this mechanical property is used in the design of double tapered, curved, and pitched cambered beams [13]. Research works determined the value of this mechanical property for various timber species [8,9,12] and engineered wood products [16,17,18]. The tensile strength perpendicular to grain can be determined by different test methods as uniaxial tensile [19,20,21,22] and three-point static bending [20,23,24]. Tannert et al. [25] investigated the tensile strength perpendicular to grain in samples of *Pinus radiata* and laminated veneer lumber made from *Pinus radiata* using various test methods. The test procedures employed were ASTM D143-21 [19], EN 408 [21], and AS/NZS 4063 [23]. The authors also determined the Weibull distribution parameters based on the results of tensile tests perpendicular to grain [25].

**Figure 1:**
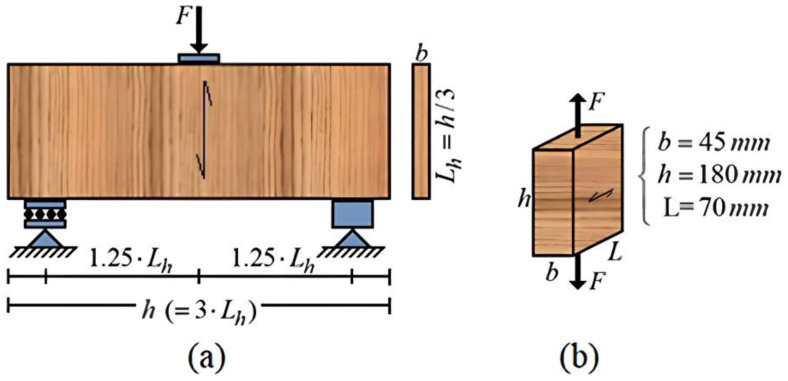
Test models for determining tensile strength perpendicular to grain (*f_t_*_,90_) transcribed from the ISO/FDIS 13910 [20]—(**a**) method A; (**b**) method B.

Code ISO/FDIS 13910 [20] establishes two test methods for determining the tensile strength perpendicular to grain (*f_t_*_,90_): three-point bending (Figure 1a, Method A) and uniaxial tensile (Figure 1b, Method B). Calculation model for obtaining of *f_t_*_,90_ is given in Equation (1) for a three-point bending test (Figure 1a).

(1) $$f_{t,90}=\left(\frac{3.75\cdot F_{ult}}{b\cdot L_{h}}\right)\cdot {\left(\frac{0.03\cdot b\cdot L_{h}^{2}}{800^{3}}\right)}^{0.20}$$

In Equation (1), *F_ult_* is the value of the applied force at failure (N), and *b* and *L_h_* the specimen thickness (smaller dimension of the cross-section in mm) and width (larger dimension of the cross-section in mm), respectively. In addition, the relation existing between length and width of the cross-section should be noted, as illustrated in Figure 1a.

The first term on the right side of Equation (1) represents the shear stress obtained from classical theory of mechanics of materials, adjusted by an amplification factor of five times. Such a factor of shear stress is due to the specimen dimensions, which cannot be considered as a one-dimensional element (long beam—classical theory of the mechanics of materials), but as a bi-dimensional element (deep beam—theory of elasticity) [26].

Since there is no established relationship between thickness and other dimensions of the specimen to be manufactured (only between length and width), its prismatic geometry, Figure 1a, may have a thickness close to one of the other two measurements [27]. Under these conditions, the surface element is now represented as a volume element. In this, the first term on the right side of Equation (1) was multiplied by a factor responsible to normalize the strength to a value similar to that obtained from a timber cube with 800 mm edges.

The *f_t_*_,90_ is determined by means of Equation (2) for uniaxial tensile test or Method B [20]. A similar equation is considered in ABNT NBR 7190-3 [22], ASTM D143-21 [19], and BS EN 408:2010+A1 [21]. In Equation (2), *F_t_*_,90,max_ is the value of the applied force at failure (N), and *A_t_*_90_ is the cross-section area in the central region of specimen (mm^2^) (Figure 1b).

(2) $$f_{t,90}=\frac{F_{t,90,\max}}{A_{t90}}$$

**Figure 2:**
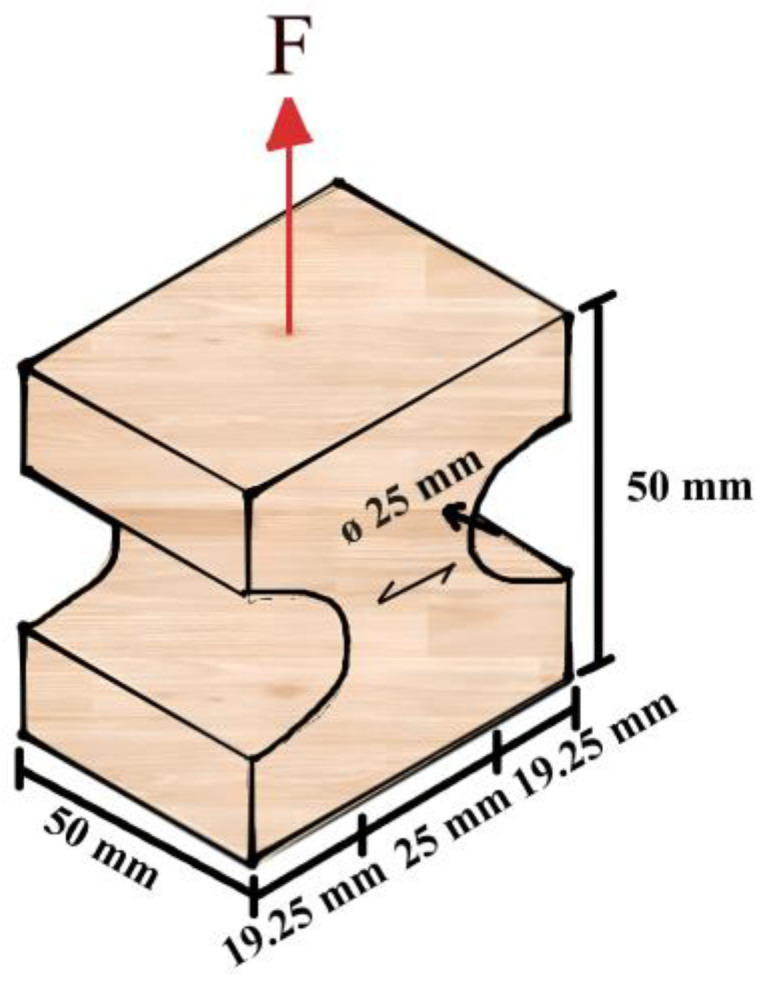
Geometry and size of specimens of the uniaxial tensile test according to ASTM D143-21 [19] and ABNT NBR 7190-3 [22].

The main distinction evidenced among ASTM D143-21 [19]; ABNT NBR 7190-3 [22]; and ISO/FDIS 13910 [20] for the uniaxial tensile test is in the format and dimensions of the specimen. Figure 2 shows the specimen format and dimensions adopted for the tensile test perpendicular to grain in accordance with ASTM D143-21 [19] and ABNT NBR 7190-3 [22].

Brazilian Code ABNT NBR 7190-4 [24] proposes the three-point bending test to determine the tensile strength perpendicular to grain of wood from a planted forest. Thus, studies are required to confirm the accuracy of the expression associated with the test methods to determine wood mechanical proprieties once the Brazilian Code ABNT NBR 7190-4 [24] is based on ISO/FDIS 13910 [20], which was developed for different wood species and edaphoclimatic conditions.

The purpose of this research was to evaluate the possible differences among the tensile strength values perpendicular to grain obtained from a three-point static bending test recommended by ISO/FDIS 13910 [20] and also by Brazilian Code ABNT NBR 7190-4 [24] (for planted forest species), as compared to the mechanical property determined by a uniaxial tensile test [19,22]. For this aim, three wood species from Brazilian planted forests (*Pinus* spp., *Eucalyptus saligna*, and *Corymbia citriodora*) were used in the experimental program.

## 2. Materials and Methods

### 2.1. Materials

In order to investigate the influence of test methods on tensile strength perpendicular to grain, three timber species from planted forests were selected for this research. The selected timber species were *Pinus* spp., *Eucalyptus saligna*, and *Corymbia citriodora*. Six boards of each species were obtained from a sawmill located in the São Carlos, Sao Paulo, Brazil, for the production of the specimens. The *Pinus* spp. trees were cut at approximately 16 years, whereas the *Eucalyptus saligna* and *Corymbia citriodora* trees were cut at approximately 20 years. Moreover, the planted forest of *Pinus* spp. was located in the Parana, Brazil, while the planted forests of *Eucalyptus saligna* and *Corymbia citriodora* were located in the Sao Paulo, Brazil. The green timber boards arrived at the laboratory clear of defects. These green timber boards were stored under ambient temperature and ambient relative humidity to achieve a moisture content close to 12% prior to sample preparation. Table 1 provides information about the timber boards used to prepare the specimens.

**Figure 3:**
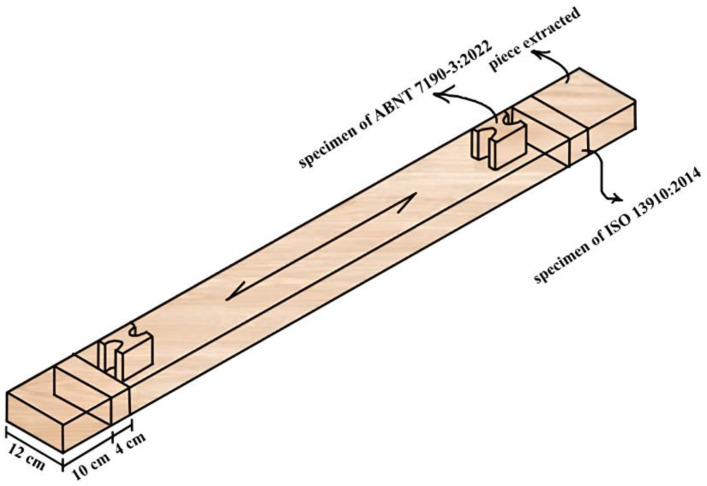
Region from extractions of specimens.

Two types of specimens were cut from timber boards for tensile tests perpendicular to grain. In total, twelve specimens of each species were prepared for each test method. This sample size was determined based on the Brazilian standard ABNT NBR 7190-3 [22], which allows for this number to characterize the strength of lesser-known species. Initially, a 10 cm length was extracted from each end of the timber boards prior to samples preparation, as shown in Figure 3. After this extraction, the samples were taken from both ends of the timber boards, as shown in Figure 3.

### 2.2. Test of Tensile Strength Perpendicular to Grain

Tensile tests perpendicular to grain were carried out according to the ABNT NBR 7190-3 [22] and ISO 13910 [20] in order to verify the difference in the strength values and failure modes of the specimens between these two methods.

#### 2.2.1. ABNT NBR 7190-3

**Figure 4:**
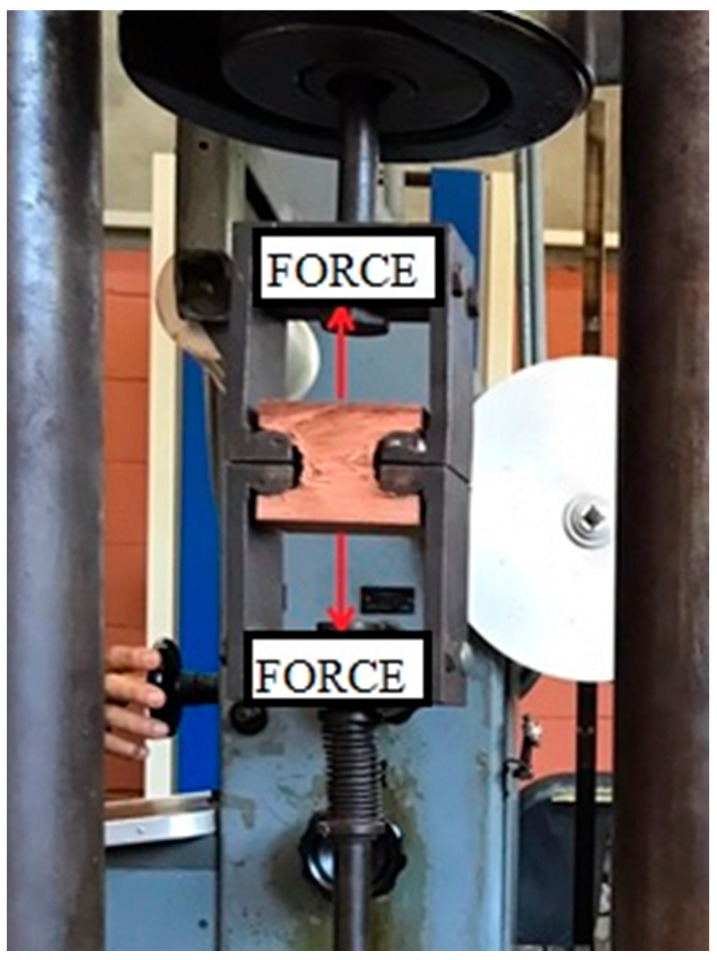
Assembly of the tensile test perpendicular to grain according to ABNT NBR 7190-3 [22].

In order to determine tensile perpendicular to grain of specimens from the three investigated planted forest species, tests were carried out as per ABNT NBR 7190-3 [22]. Experimental tests were performed at room temperature (RT). A universal testing machine (AMSLER) with a force capacity of 250 kN was used to apply the tensile force. The experimental setup is represented in Figure 4. The force was applied at a constant rate of about 2.5 MPa/min until specimen failure. Tensile strength perpendicular to grain was calculated using Equation (2).

#### 2.2.2. ISO 13910

**Figure 5:**
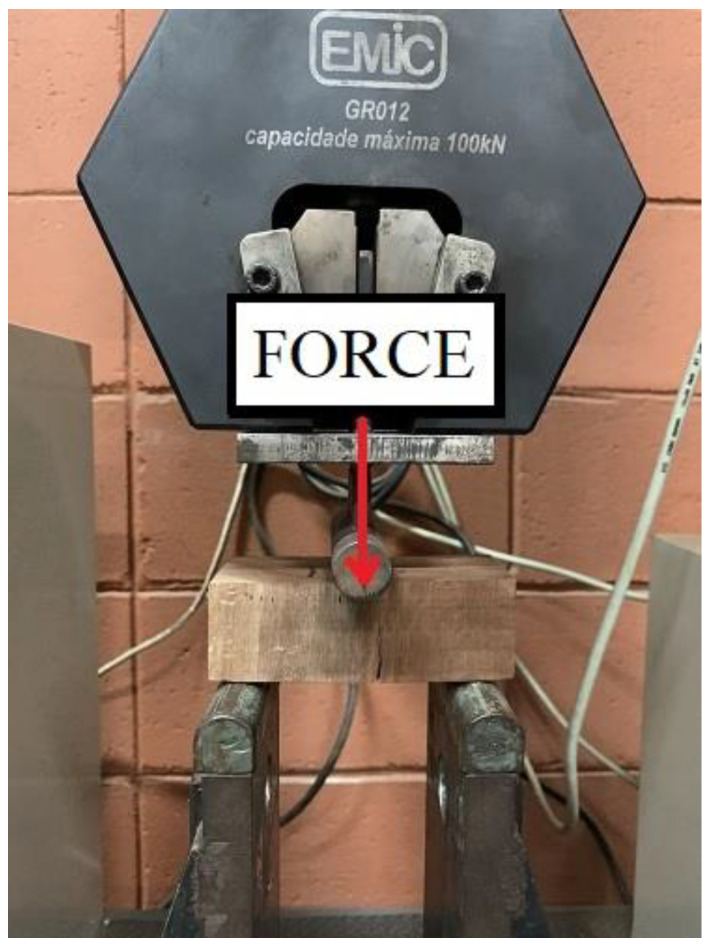
Assembly of the three-point bending test according to ISO 13910 [20].

In order to determine tensile perpendicular to grain of specimens from three planted forest species, tests were carried out as per ISO 13910 [20]. According to ISO 13910 [20], the tests shall be performed on specimens obtained from the full cross-section of the timber board. The experimental tests were performed at RT. The EMIC universal testing machine with a load capacity of 30 kN was used to apply the bending load. The force was applied at a constant rate of 1 mm/min until specimen failure. The experimental setup is represented in Figure 5. The tensile strength perpendicular to grain was calculated using Equation (1).

### 2.3. Moisture Content

The moisture content was determined for each specimen tested. First, the specimen weight was obtained with a precision of 0.01 g immediately prior to testing. After the tests, the specimens were placed in an oven at a temperature of 105 °C until a constant mass was reached. According to ABNT NBR 7190-3 [22], the constant mass is achieved when the variation between two weight measurements within a 6 h interval is less than 0.5%. The moisture content was calculated using Equation (3).

(3) $$U=\frac{m_{m}-m_{o}}{m_{o}}\times 100$$

where *U* is the moisture content of the specimen at the time of test (%), *m_m_* is the initial mass at the time of test (g), and *m_o_* is the oven-dry mass (g).

### 2.4. Tensile Strength Perpendicular to Grain with Moisture Content of the 12%

Brazilian Code [14] defines a 12% wood moisture content as a pattern for structural design. Therefore, according to the cited code, the strength value obtained in the characterization test about the moisture content of the specimen must be corrected to a moisture content of 12%. Thus, the tensile strength perpendicular to grain (*f_t_*_,90,*U*_) of each specimen was corrected to the moisture content of 12% (*f_t_*_,90,12_) using Equation (4).

(4) $$f_{t,90,12}=f_{t,90,U}\cdot \left[1+\frac{3\cdot \left(U-12\right)}{100}\right]$$

where *f_t_*_,90,12_ is the tensile strength perpendicular to grain corrected for the moisture content of the 12% (MPa); *f_t_*_,90,*U*_ is the tensile strength perpendicular to grain at the time of test (MPa); and U is the moisture content of the specimen at the time of test (%).

### 2.5. Statistical Analyses

The corrected strength results (Section 2.4) for uniaxial tensile and three-point bending were compared using analysis of variance (ANOVA) with a 5% significance level to identify possible differences.

Adjustments in Equation (1) were proposed due to the significant difference in strength obtained from both test methods. Thus, regression models were considered in a way to evaluate the possibility of improvements in the precision of Equation (1). The mean absolute percentage error (*MAPE*—Equation (5)) was used to compare the results obtained using models adapted to Equation (1) (*Y_predict_*) with the results of *f_t_*_,90_ determined by a uniaxial tensile test (*Y_data_*), where n is the number of specimens considered in generations of models.

(5) $$MAPE(\%)=100\cdot \frac{1}{n}\cdot \sum_{i=1}^{n}\left\|\frac{Y_{predict_{i}}-Y_{data_{i}}}{Y_{data_{i}}}\right\|$$

## 3. Results and Discussion

**Figure 6:**
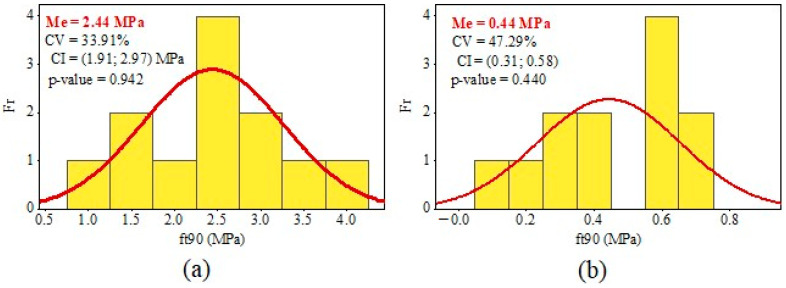
Results of tensile strength values perpendicular to grain (*f_t_*_,90_) from *Pinus* spp. wood: (**a**) uniaxial tensile; (**b**) three-point bending.

**Figure 7:**
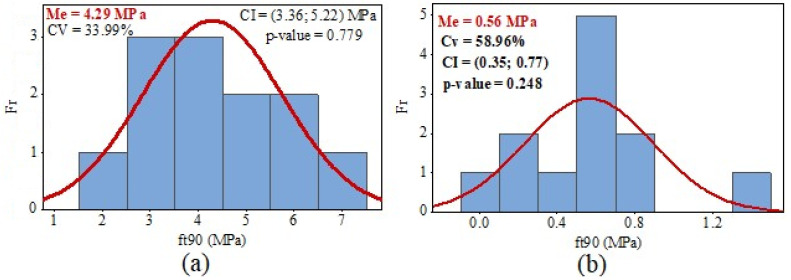
Results of tensile strength values perpendicular to grain (*f_t_*_,90_) from *Eucalyptus saligna* wood: (**a**) uniaxial tensile; (**b**) three-point bending.

**Figure 8:**
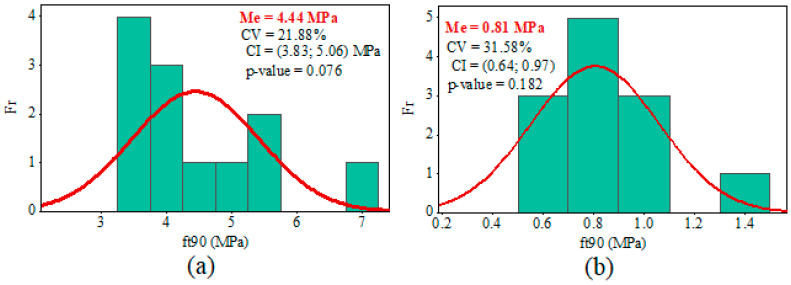
Results of tensile strength values perpendicular to grain (*f_t_*_,90_) from *Corymbia citriodora* wood: (**a**) uniaxial tensile; (**b**) three-point bending.

Mean values (Me), coefficients of variation (CV), confidence intervals for the mean (CI, at a 95% confidence level), frequency histograms (Fr), and the *p*-values from the Anderson–Darling normality test (at a 5% of significance) regarding the *f_t_*_,90_ values obtained from the three-point bending test (Equation (1)) and uniaxial tensile test (Equation (2)) are shown in Figure 6, Figure 7 and Figure 8.

The results of the Anderson–Darling normality test (5% of significance) are greater than the significance level, as shown in Figure 6, Figure 7 and Figure 8. Thus, the results of the ANOVA and CI for the Me are valid because the distribution of *f_t_*_,90_ values for test methods and, consequently, the wood species, were considered normal.

The CVs obtained from the uniaxial tensile tests for determining the tensile strength perpendicular to grain were lower than the CVs from the three-point bending tests for all the species studied. However, Tannert et al. [25] obtained higher coefficients of variation in uniaxial tensile tests than in there-point bending tests. The CV for *Pinus radiata* samples was 34% for the uniaxial tensile test and 16% for the there-point bending test [25]. Moreover, Tannert et al. [25] also showed that CV for laminated veneer lumber samples from *Pinus radiata* was 37% for the uniaxial tensile test and 34% for the there-point bending test.

The difference between the mean values of *f_t_*_,90_ obtained from the three-point bending tests (Equation (1)) and uniaxial tensile tests (Equation (2)) was significantly elevated (*p*-value of the ANOVA <0.05). As a result, the difference in the mean strengths of the *Eucalyptus saligna* samples was 766.07%. This discrepancy was the highest observed among all the species investigated. Furthermore, the *Corymbia citriodora* samples differed by 548.15% between the two test methods, whereas *Pinus* spp. samples differed by 554.55%. However, the number of specimens per sample was too small; therefore, additional tests should be performed to verify the discrepancy in strength values between the two testing methodologies.

Some adaptations to Equation (1) from Code ISO 13910 [20] were carried out due to the noted differences in the results between the two calculation methods for the determination of the *f_t_*_,90_. In such adaptations, the values from the uniaxial tensile tests were regarded as the reference values for *f_t_*_,90_. First, an attempt was made to determine the coefficients *α*_0_, *α*_1_, and *α*_2_ from Equation (6) using a non-linear regression model (Marquardt algorithm, 2000 iteration and tolerance of 1.10^−3^). For this purpose, sampling results from the three wood species examined were combined.

(6) $$f_{t,90}=\left(\frac{\alpha_{0}\cdot 3.75\cdot F_{ult}}{b\cdot L_{h}}\right)\cdot {\left(\frac{\alpha_{1}\cdot 0.03\cdot b\cdot {L_{h}}^{2}}{800^{3}}\right)}^{0.20\cdot \alpha_{2}}$$

As a result, the coefficients *α*_i_ for Equation (6) consisted of *α*_0_ = 0.886, *α*_1_ = 0.001, and *α*_2_ = 0.186. These coefficients *α*_i_ resulted in a mean absolute percentage error (*MAPE*) of 32.85%. An analysis was carried out to assess the influence of the coefficients *α*_i_ in Equation (6). The new values of *α*_0_ and *α*_1_ were 3.301 and 10, respectively, with an associated *MAPE* of 33.13% after removing the coefficient *α*_2_. On the other hand, the new values of *α*_0_ and *MAPE* were 5.233 and 33.14%, respectively, after removing the coefficients *α*_1_ and *α*_2_. As can be verified, the adjustment that takes into account only *α*_0_ resulted in the same order of *MAPE* when compared to the models containing two (*α*_0_ e *α*_1_) and three coefficients (*α*_0_, *α*_1_ e *α*_2_). Therefore, determination of tensile strength perpendicular to grain can be estimated just with the adoption of the coefficient *α*_0_ = 5.233, Equation (7). Moreover, the *MAPE* obtained using Equation (1) was 82.18%. Thus, it is necessary to calibrate the equation proposed by ISO/FDIS 13910 [20] and ABNT NBR 7190-4 [24].

(7) $$f_{t,90}=\left(\frac{\overset{\alpha_{0}}{\overset{︷}{5.233}}\cdot 3.75\cdot F_{ult}}{b\cdot L_{h}}\right)\cdot {\left(\frac{0.03\cdot b\cdot {L_{h}}^{2}}{800^{3}}\right)}^{0.20}$$

**Figure 9:**
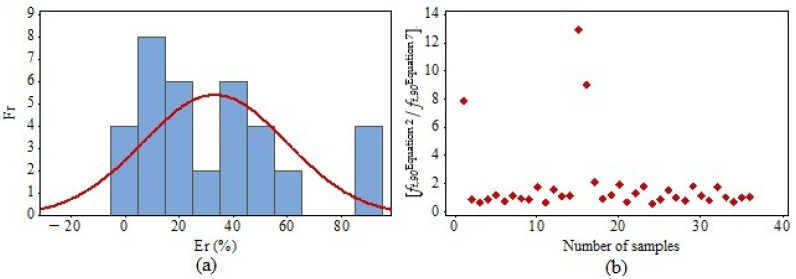
(**a**) Frequency histogram (Fr) of error committed with the use of the Equation (7); (**b**) ratio among the *f_t_*_,90_ [*f_t_*_,90_^Equation (2)^] obtained by Equation (2), and the *f_t_*_,90_ [*f_t_*_,90_^Equation (7)^] obtained by Equation (7) [*f_t_*_,90_^Equation (2)^/*f_t_*_,90_^Equation (7)^].

The frequency histogram (Fr) of the errors committed using Equation (7) is shown in Figure 9a, whereas Figure 9b presents a scatter plot of the values obtained from the ratio of *f_t_*_,90_ determined by Equation (7) (three-point bending) and Equation (2) (uniaxial tensile).

Approximately 50% (18 of 36) of the results estimated in Equation (7) have errors less than or equal to 20%, as shown in Figure 9a. In addition, 15 of the 36 estimated values (41.67%) were less than 1; therefore, 58.33% of the results were estimated in favor of safety. Table 2 shows the results (Me and CV) of *f_t_*_,90_ obtained using Equation (7), as well as the corresponding *MAPE*. Moreover, the wood species evaluated were considered isolated.

The ratios of *f_t_*_,90_ values obtained using Equation (7) (three-point bending) to those determined by Equation (2) (uniaxial tensile) for the species of *Pinus* spp., *Eucalyptus saligna*, and *Corymbia citriodora* were 0.91, 0.70 and 0.96, respectively.

**Figure 10:**
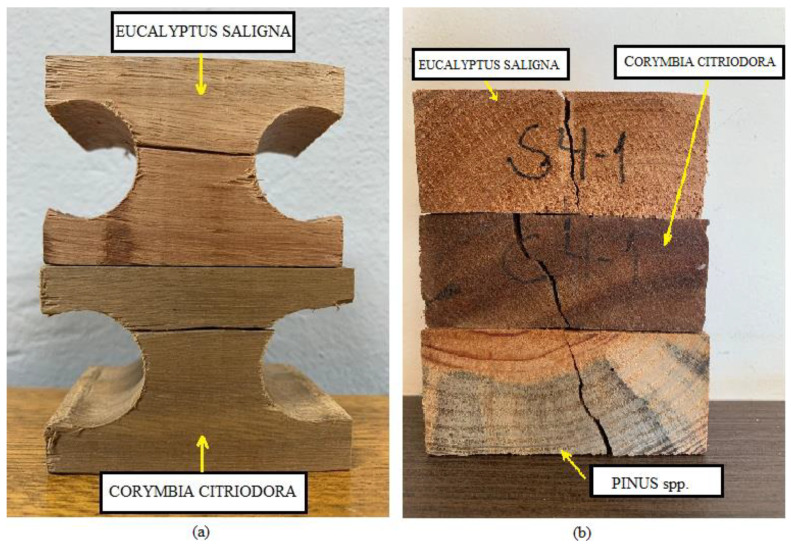
(**a**) typical failure in specimens submitted the axial test of tensile perpendicular to grain based on ABNT NBR 7190-3 [22]; (**b**) crack in medullary rays in the specimens from ISO/FDIS 13910 [20].

Figure 10a shows the failure modes of the specimens tested according to ABNT NBR 7190-3 [22]. Brittle fracture was the predominant failure mode observed in each sample for this test method. In addition, the grains within the fracture surface broke almost at the same time. The crack initially occurred in medullary rays at the edge where the tension stress is maximum in the bending test samples, as shown in Figure 10b. Then, the crack propagated vertically until specimen rupture.

## 4. Conclusions

The influence of test method on determination of tensile strength perpendicular to grain of timber for civil construction was presented. For this purpose, two different test methods were analyzed: uniaxial tensile and three-point bending. The main conclusions for this research are:

- Tensile strength values perpendicular to grain (*f_t_*_,90_) of the samples obtained from the uniaxial tensile test were significantly higher (around 5.55 and 7.7 times) than the values determined by the three-point static bending test.
- Correction factor for the equation from Code ISO 13910 [20], used to estimate *f_t,90_* based on three-point bending test, was the term that had a significant impact on the obtained results. This impact resulted in much lower bending strength values compared with those obtained from the uniaxial tensile test.
- The values of the mean absolute percentage error were very similar to the results obtained with the adoption of the proposed coefficients for Equation (1). The model with a smaller adaptation of the equation from Code ISO/FDIS 13910 [20] was adopted in this research. Therefore, the coefficient 5.233, inserted into the term [(3.75·Fult)/b·Lh] of the equation from Code ISO/FDIS 13910 [20] and Brazilian Code ABNT NBR 7190-4 [24], produced results more similar when compared with the uniaxial tensile test results.
- The disparity in outcomes can be attributed to the different stress conditions inherent to each testing method. In the three-point bending test, wood grains and parenchyma cells are subjected to both normal and tangential stresses. In contrast, only normal stress is applied in wood grains in the uniaxial tensile test.
- In the design of timber structures, the tensile strength perpendicular to grain affects the dimensions of double tapered, curved, and pitched cambered beams. Thus, the design of these beams becomes more conservative by taking into account the most unfavorable conditions in the structural design (tensile strength obtained from the three-point static bending test).

The number of specimens was relatively small for each sample, therefore, additional tests with other species should be performed to verify the discrepancy in tensile strength values perpendicular to the grain between the two testing methodologies.

## Figures and Tables

**Table 1:** Features and nominal dimensions of timber boards.

Wood Species	Number of Boards	Width (mm)	Thickness (mm)	Length (cm)	Mean Density (kg/m^3^)
*Pinus* spp.	6	120	50	200	589.96 (64.3) ^1^
*Eucalyptus saligna*	6	120	50	200	738.21 (114.6)
*Corymbia citriodora*	6	120	50	200	916.64 (40.5)

^1^ Values in brackets correspond to the standard deviation.

**Table 2:** Results of *f_t_*_,90_ (MPa) obtained with the Equation (7) (adaptation of the Equation (1)).

Wood Species	Equation (7)	Equation (2)	*MAPE* (%)
*Pinus* spp.	2.23 (46.27%)	2.44 (33.91%)	34.11
*Eucalyptus saligna*	3.02 (57.59%)	4.29 (33.99%)	44.82
*Corymbia citriodora*	4.25 (31.23%)	4.44 (21.88%)	20.48

## Data Availability

The raw data supporting the conclusions of this article will be made available by the authors on request.
